# Metabolic Engineering of Microbial Cell Factories for Biosynthesis of Flavonoids: A Review

**DOI:** 10.3390/molecules26154522

**Published:** 2021-07-27

**Authors:** Hanghang Lou, Lifei Hu, Hongyun Lu, Tianyu Wei, Qihe Chen

**Affiliations:** 1 Department of Food Science and Nutrition, Zhejiang University, Hangzhou 310058, China; louhanghang@zju.edu.cn (H.L.); luhongyun@zju.edu.cn (H.L.); 21913067@zju.edu.cn (T.W.); 2 Hubei Key Lab of Quality and Safety of Traditional Chinese Medicine & Health Food, Huangshi 435100, China; lfyhu@hotmail.com

**Keywords:** flavonoids, metabolic engineering, co-culture system, biosynthesis, microbial cell factories

## Abstract

Flavonoids belong to a class of plant secondary metabolites that have a polyphenol structure. Flavonoids show extensive biological activity, such as antioxidative, anti-inflammatory, anti-mutagenic, anti-cancer, and antibacterial properties, so they are widely used in the food, pharmaceutical, and nutraceutical industries. However, traditional sources of flavonoids are no longer sufficient to meet current demands. In recent years, with the clarification of the biosynthetic pathway of flavonoids and the development of synthetic biology, it has become possible to use synthetic metabolic engineering methods with microorganisms as hosts to produce flavonoids. This article mainly reviews the biosynthetic pathways of flavonoids and the development of microbial expression systems for the production of flavonoids in order to provide a useful reference for further research on synthetic metabolic engineering of flavonoids. Meanwhile, the application of co-culture systems in the biosynthesis of flavonoids is emphasized in this review.

## 1. Introduction

**Figure 1:**
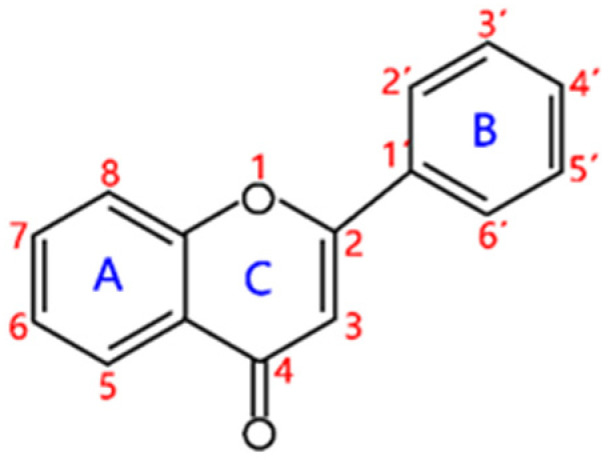
General structure of flavonoids.

Flavonoids are a kind of secondary metabolite produced by plants in the process of long-term natural selection. They are widely found in vegetables, fruits, and medicinal plants. Structurally, they possess a C6–C3–C6 carbon framework, which is formed by connecting two aromatic rings (rings A and B) through a heterocyclic ring that contains three carbon atoms (ring C) [1] (Figure 1). Thus far, about 10,000 flavonoids have been discovered, which comprise one of the largest families of natural products [2]. There are two forms of flavonoids in nature, one occurs in aglycone form in plants, and the other combines with sugar to form glycosides [3]. Common sugars attached to flavonoids include D-glucose, D-galactose, L-rhamnose, D-xylose, D-glucuronic acid, sophorose, rutinose, tangerine peel sugar, and gentianose [3,4]. Flavonoids normally occur in plants as O-glycosylated derivatives [5], and sugars are mostly attached to the hydroxyl groups at position 3 of the C-ring and positions 5 and 7 of the A-ring. However, some plants, such as maize, wheat, and rice, also produce C-glycosylated flavonoids [5]. These flavonoids play important roles in plants, animals, and humans. In plants, flavonoids are responsible for flower color and aroma and attract pollinators to disperse fruit and help plants propagate [6]. In addition, they also help plants combat various biotic and abiotic stresses, including ultraviolet protection in leaves, protection from microbial infections and herbivores, and defending from physical and radical damage [7,8]. As a kind of phytochemicals, flavonoids cannot be synthesized by humans or animals. Therefore humans generally absorb flavonoids from fruits, vegetables, legumes, and other foods, and flavonoids are thought to have health-promoting properties. Most studies have found that flavonoids have multiple physiological and pharmacological activities, such as antioxidative, anti-inflammatory, anti-mutagenic, anti-cancer, antiviral, antibacterial, and protecting the heart and cerebrovascular vessels [6,9,10]. Therefore, flavonoids show extensive applications in functional foods and some medicine. In fact, some flavonoid extracts, such as Masquelier’s grape seed extract, have been sold as dietary supplements [11].

The wide application of flavonoids has led to their increasing demand. At present, flavonoids are generally extracted from plants. However, plants have a long culture period, and their growth is also limited by the temperature and season. Additionally, the process of extraction and purification could increase the cost of production and lead to the loss of the product and the reduction of product activity [12]. Therefore, it is a huge challenge to produce these compounds on a large scale through plant extraction to meet medical and nutritional needs [13]. The total synthesis of some flavonoids has been realized by chemical approaches; however, this method required some extreme reaction conditions, such as high temperature, strong acid, strong base, and the use of heavy metal, which is unfriendly to the environment and hindered its scale-up and commercial application. In recent years, the production of flavonoids by microbial cell factories has attracted wide attention with the development of metabolic engineering tools, the in-depth studies on the synthesis pathway of flavonoids and available substrates [7,12,14]. Engineering the whole biosynthetic pathway to produce flavonoids in microorganisms has many advantages, such as a short production cycle, low waste production, low energy requirements, and mass production. Some microorganisms, such as *Escherichia coli* (*E. coli*), *Saccharomyces cerevisiae* (*S. cerevisiae*), *Yarrowia lipolytica (Y. lipolytica)*, grow faster, and various genetic manipulations of this species and metabolic modifications have been studied in detail, so it has become possible to use these microorganisms as hosts for the production of flavonoids [2,15,16,17]. In a previous study, the production of flavonoids by genetically engineered bacteria had not been achieved, and one of the barriers to produce these compounds was the difficulty in the expression of active C4H. In 2003, a 4CL (4-coumarin-CoA ligase) was discovered in the gram-positive filamentous bacterium *Streptomyces coelicolor* [18]. The enzyme not only activated cinnamic acid to cinnamoyl-CoA but also activated 4-coumaric acid to 4-coumaroyl-CoA. The use of the enzyme would bypass the C4H step for the production of pinocembrin chalcone from phenylalanine via the phenylpropanoid pathway [18]. In this study, the synthesis of flavonoids in microorganisms was achieved for the first time when PAL (phenylalanine ammonia lyase), CHS (chalcones synthase), and 4CL (4-coumarin-CoA ligase) were engineered in *E. coli* to produce naringenin and pinocembrin [18]. Since then, more and more enzymes related to flavonoid synthesis from different plants and microorganisms have been discovered and well-characterized [7], and meanwhile, various flavonoid pathways have been reconstituted in microbial species, including *E. coli*, *S. cerevisiae*, *Streptomyces albus* (*S. albus*), and *Streptomyces coelicolor* (*S. coelicolor*), to produce naringenin, eriodyctiol, pinocembrin, anthocyanins, scutellarein, baicalein, myricetin, kaempferol, quercetin, liquiritigenin, resokaempferol, fisetin, and so forth [13,16,17,19,20]. However, some problems, including the limitation of the availability of pathway genes, the instability of engineered strains, and the low yield of products in the synthesis process, still hinder the microbial production of flavonoids [1]. Several strategies have been employed to solve these problems, including the optimization of culture conditions, modular co-culture technology, enhancement of malonyl-CoA, exploration of different substrates, optimization of functional expression of plant-derived P450 enzymes and fine-tuning the synthetic pathway using an iterative high-throughput screening method [21,22]. Among them, microbial co-cultures are gradually being investigated in-depth due to their many advantages over mono-cultures in terms of eliminating pathway bottlenecks and improving metabolic efficiency. In this review, the structural features, classification of flavonoids, and biosynthesis of flavonoids in plants are introduced in detail. Then, the biosynthesis of flavonoids in mono-cultures and co-cultures is discussed.

## 2. Structural Features and Classification

According to structural differences, such as the variation of the C-ring and the position of the B-ring, flavonoids can be classified into flavones, flavonols, isoflavones, anthocyanins, chalcone, aurones, neoflavanoids, and their dihydrogen derivatives [6,7] (Table 1). For example, the structural feature of chalcones and dihydrochalcones is the absence of a C-ring in the basic flavonoid skeleton structure [7]. The C-ring of aurones is a five-membered carbon ring [7]. The C-ring of other flavonoids is mainly a six-membered carbon ring. Flavonoids in which a B-ring is connected to position 3 of a C-ring are called isoflavonoids, while those in which the B-ring is connected to position 4 are called neoflavonoids [7]. The B-ring of other flavonoids is mainly linked to position 2 of the C-ring [7]. Meanwhile, the types of flavonoids become diversified due to the chemical modifications by the differential placement of phenolic hydroxyl groups and other functional group modifications, such as methylation, methoxylation, glycosylation, acylation, malonylation, and so on [23].

### 2.1. Flavones and Flavanones

Structurally, flavones have a ketone in position 4 of the C-ring and a double bond between the second and third carbon atoms. The B-ring of flavones is linked in position 2 of the C-ring. These compounds are widely found in some plants encompassing celery, parsley, red peppers, chamomile, mint, and ginkgo biloba [6]. In particular, some natural flavones simultaneously have hydroxyl groups in positions 5 and 7 of the A-ring, and the 3′ and 4′ sites of the B-ring often have hydroxyl or methoxy groups. Their representative compounds are apigenin, luteolin, baicalein, and tangeritin (Table 1). Flavones also are present as glycosides, and rhamnose or glucose is generally attached to position 7 of the A-ring in most flavone glycosides.

Flavanones, also called dihydroflavones, are dihydrogen derivatives of flavones. They have the saturated C-ring, which has no conjugation between both rings A and B [6]. There is a chiral carbon atom in flavanones, which are optically active. They are widely distributed in some higher plant families, such as *Compositae*, *Leguminosae*, and *Rutaceae* [24]. Flavanones derived from the citrus fruits have been widely investigated due to interesting pharmacological effects, such as antioxidant, anti-inflammatory, blood lipid-lowering, and cholesterol-lowering agents, in which hesperitin, naringenin, and their derivatives are characteristic compounds [24] (Table 1). The main source of hesperitin and its derivative are sweet orange, tangelo, lemon, and lime, while naringenin and its derivative mainly come from grapefruit and sour orange [24,25]. Naringenin is a precursor of other classes of flavonoids, such as isoflavones, flavanols, anthocyanins, flavonols, and flavones [26,27]. The juice and peel of citrus fruits have bitterness because of the presence of these compounds [28].

### 2.2. Flavonols and Flavanonols

Flavonols are one of the important subgroups of flavonoids. They are the most abundant and widely distributed subgroup of flavonoids in comparison to other flavonoids [6]. Compared with flavones, flavonols have a hydroxyl group in position 3 of the C-ring, which may also be attached to O-glycosides [6]. Among the flavonols that have been discovered, there are more than ten kinds of sugars bound to flavonols, such as glucose, rhamnose, galactose, sophorose, and rutabiose, most of which are monosaccharides, but also disaccharides or trisaccharides. Some fruits, vegetables, and teas, such as onions, kale, lettuce, tomatoes, broccoli, apples, plums, grapes, and berries, are the major sources of flavonols. The most studied flavonols are kaempferol, quercetin, myricetin, and rutin (Table 1).

Flavanonols, also called dihydroflavonol, are dihydrogen derivatives of flavonols, which are the 3-hydroxy derivatives of flavanones. They have a positive effect on human health as they prevent the accumulation of free radicals and have anti-inflammatory, antioxidant, and antidiabetic activities [29]. Dihydroflavonols exist widely in some plants, such as *Pseudotsuga menziesii*, and *Larix gmenlinii* [30,31]. Aromadendrin, distylin, dihydrokaempferol, and dihydroquercetin are examples of this class of flavonoids (Table 1).

### 2.3. Flavanols

Flavanols are a complex subclass of flavonoids, which include a variety of monomeric, oligomeric and polymeric compounds. The monomeric forms of flavanols are catechins, mainly encompassing catechin (C), epicatechin (EC), epigallocatechin (EGC), epicatechin gallate (ECG), and epigallocatechin gallate (EGCG), while the oligomeric or polymeric compounds are proanthocyanidins [32]. Among them, the polymers composed exclusively of catechin or epicatechin are called procyanidins [33]. Flavanols are abundant in cocoa, green tea, grapes, apples, and red wine, and cocoa flavanols are extensively studied owing to their higher flavanol content than other flavonoid-containing products, such as tea and wine [34,35]. These compounds have been observed to exert beneficial effects on reducing the risk of suffering from chronic diseases (cardiovascular diseases, metabolic disorders, cancer) [36]. From a structural point of view, flavanols lack a ketone oxygen in position 4 of the C-ring and the double bond between positions 2 and 3 of the C-ring, but they have multiple hydroxyl groups on A, B, and C-rings. In particular, flavanols in which there is a hydroxyl group in position 3 of the C-ring are called flavan-3-ols (also known as catechins), while flavanols are also referred to as flavan-3,4-diols, as position 3,4 of the C-ring has a hydroxyl group, respectively [12]. Catechin, elephantorrhizol, guibourtinidol, and epirobinetinidol belong to flavan-3-ols, and leucofisetinidin, leucocyanidin, melacacidin, and leucopeonidin belong to flavan-3,4-diols (Table 1).

### 2.4. Anthocyanidins

Anthocyanidins belonging to the flavonoid family are one of the most common water-soluble pigments in nature. In plants, anthocyanidins are always glycosylated and exist in the form of anthocyanins, and they are responsible for the vibrant color (red, orange, blue, purple) of fruits, flowers, leaves, vegetables, and grains [37]. The color of these compounds depends not only on pH, but is also affected by methylation or acylation at the hydroxyl groups on the A and B-rings [38]. Usually, they are used to color food for substitution of synthetic colorants in the food industry. Meanwhile, anthocyanidins are beneficial to human health because of their antioxidant, anti-obesity, anti-inflammatory, and anti-carcinogenic properties [37]. Anthocyanidins and anthocyanins are unstable and easily affected by many factors, such as temperature, oxygen, pH, light, metal ions, and enzymes [39]. Structurally, anthocyanidins have the basic structure of flavylium ion but are not equipped with a ketone oxygen in position 4 of the C-ring [37]. Cyanidin, delphinidin, malvidin, peonidin, pelargonidin, and petunidin are the most common anthocyanidins distributed in plants (Table 1).

### 2.5. Chalcone

Unlike many flavonoids, chalcones are open-chain flavonoids with two aromatic rings linked by a three-carbon enone moiety [40]. Chemically, chalcones possess a 1,3-diphenyl-2-propenon core structure, which is divided into hydroxychalcones, methoxychalcones, aminochalcones, arylchalcones, alkylchalcones, nitrogenous chalcones, and others according to the different substituents [40,41]. Chalcones are biogenetic precursors of flavones. Chalcone is an important plant pigment, and it can provide a yellow color to the petals of some medicinal plants due to a very conjugated system in its skeleton [42]. Chalcones are abundantly present in strawberries, bearberries, tomatoes, pears, and certain wheat products [6]. Naringenin chalcone, pinocembrin chalcone, echinatin, and licochalcone A are prominent members of the chalcone family (Table 1). Chalcones have attracted wide attention because of their numerous nutritional and pharmacological activities, such as antibacterial, antinociceptive, anticonvulsant, and anti-inflammatory. The dihydro derivatives of chalcones are dihydrochalcones, some examples of which are neohesperidin dihydrochalcone, phloridzin, phloretin, and naringin dihydrochalcone (Table 1). Neohesperidin dihydrochalcone (NHDC), widely extracted from natural citrus plants, is an important dihydrochalcone, and it has been widely studied as a food additive because of its sweet taste [43].

### 2.6. Aurones

Aurones represent a minor class of flavonoids, which was discovered in 1943 and named “aurone” (from Latin *aurum*, “gold”) by Bate-Smith and Geissman in 1951 [44]. Structurally, aurones contain a benzofuranone core and a phenyl group linked by a carbon-carbon exocyclic double bond [44]. They are regarded as isomers of flavones and isoflavones and also can be synthesized by the oxidative cyclization of 2′-hydroxychalcones [45]. Aurones occur in flowers of *Asteraceae*, *Plantaginaceae*, *Oxalidaceae*, *Gesneriaceae*, *Rosaceae* and others, tree heartwoods of *Fabaceae*, *Anacardiaceae*, and *Rhamnaceae*, seeds and roots of non-tree *Fabaceae*, *Moraceae*, and other families [44]. Currently, more than one hundred compounds have been isolated from plants and fully investigated, including their glycosylated derivatives, auronols, and dimers. It is worth noting that aurones not only have advantages for plants, such as flower coloration and pollination, photoprotection, and anti-infection, but also show a wide range of biological activity, including antiviral, antibacterial, antifungal, anti-inflammatory, antitumor, antimalarial, antioxidant, neuropharmacological activities, and so on [46].

### 2.7. Isoflavonoids

Isoflavonoids are a distinctive subgroup of flavonoids existing in abundance in plants belonging to the families *Fabaceae* and *Iridaceae* [47]. Some isoflavonoids have also been reported to be isolated from fungi and actinomycetes [48,49]. Isoflavonoids possess a molecular framework formed by 15 carbon atoms with the general structure of C6–C3–C6, but unlike other flavonoids (such as flavones, flavonols, flavanones), the B-ring of isoflavonoids is attached to position 3 of the C-ring. Isoflavonoids are divided into isoflavones, isoflavanones, isoflavans and so on [7]. Because their structure is similar to estrogen, they can interact with estrogen receptors and exhibit weak estrogenic activity, so isoflavonoids are sometimes called phytoestrogens [50]. In addition, isoflavonoids are extensively reported for their other biological and pharmacological activities, including antidiabetic, antibacterial, antifungal, antiviral, antitumor, anti-inflammatory, and antiaging [48]. Examples of most studied isoflavonoids that have been displayed include daidzein, genistein, biochanin A, glycitein, and formononetin (Table 1).

### 2.8. Neoflavonoids

Neoflavonoids belong to a further small subclass of flavonoids, and they also have the general structure of C6–C3–C6 with the B-ring connected to position 4 of the C-ring. Neoflavonoids contain neoflavone (4-arylcoumarin), 3,4-dihydro-4-arylcoumarins, and neoflavene (4-arylchromene) [7]. Neoflavonoids are present in a wide variety of plants from families *Passifloraceae*, *Asteraceae*, *Clusiaceae*, *Leguminosae*, *Rubiaceae*, and *Rutaceae* [51]. Calophyllolide isolated in 1951 from extracts of *Calophyllum inophyllum* seeds was the first new neoflavonoid from natural sources [52]. Dalbergin is the most common and widely distributed neoflavone in plants [52].

### 2.9. Biflavonoids

Structurally, biflavonoids are characterized as dimeric flavonoids, which generally comprise dimers of flavone–flavone, flavone–flavonone, flavonone–flavonone, chalcone–isoflavone, and flavanone–chalcone [53]. The individual flavones in the dimer are directly interconnected by C‒C or C‒O‒C bonds [53]. Amentoflavone and robustaflavone belong to biflavonoids connected by the C–C bond, while ochnaflavone and hinokiflavone are examples of biflavonoids connected via the C–O–C bond [54]. Biflavonoids are found in plants such as *Selaginella tamariscina*, *Ginkgo biloba*, *Cephalotaxus koreana*, *Nandina domestica*, and *Lonicera japonica* [54]. The first biflavone isolated from natural sources was ginkgentin. It was isolated in 1929 from *Ginkgo biloba* L. as a yellow pigment [55]. Biflavonoids exhibit extensive pharmacological properties, including anti-inflammatory, antioxidant, inhibitory activity against phospholipase A2 (PLA2), and antiprotozoal activity [55].

## 3. Biosynthesis of Flavonoids in Plants

**Figure 2:**
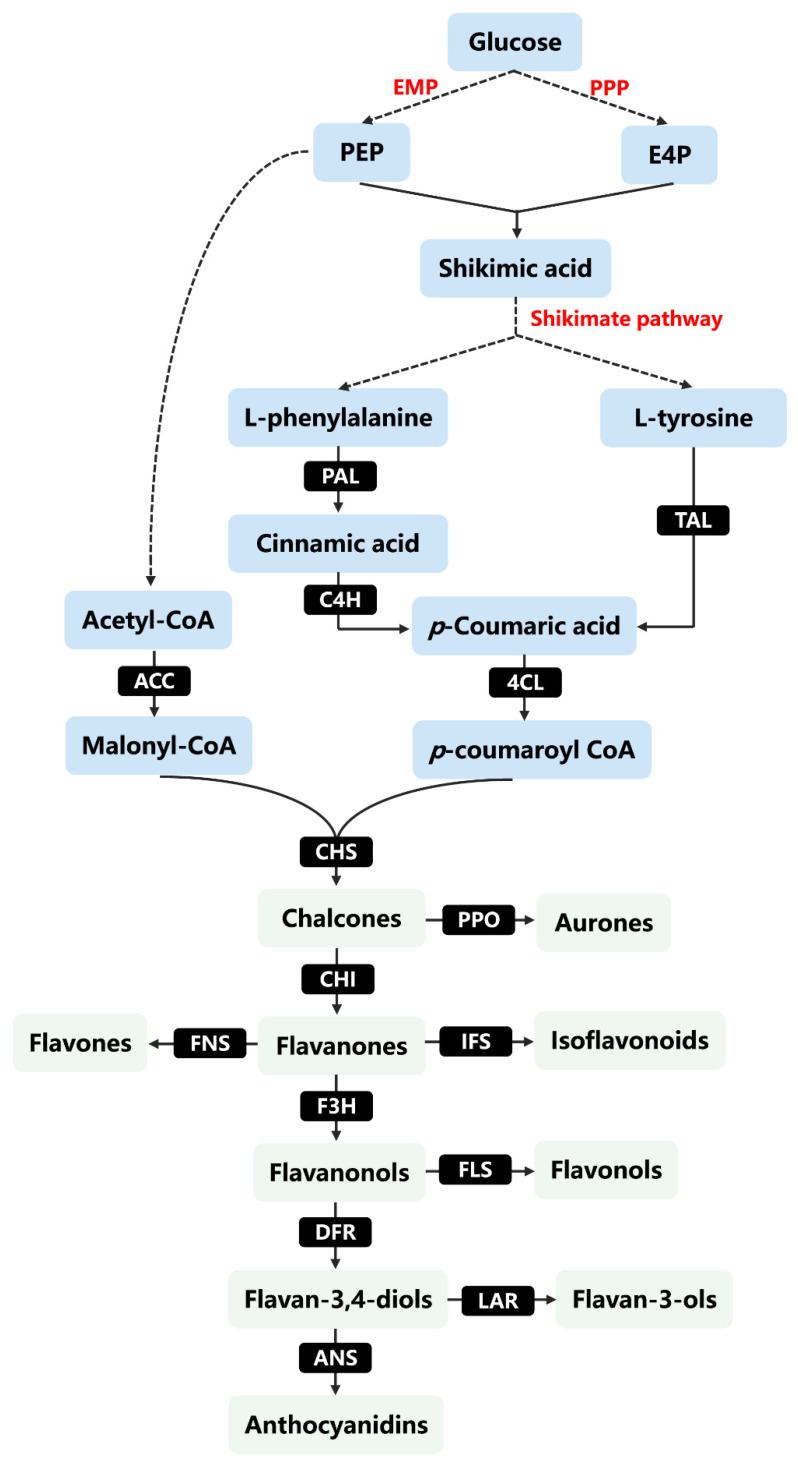
Biosynthesis pathway network of flavonoids in plants. EPM: glycolytic pathway; PPP: pentose phosphate pathway; PEP: phosphoenolpyruvate; E4P: erythrose 4-phosphate; PAL: phenylalanine ammonia lyase; C4H: cinnamate 4-hydroxylase; TAL: tyrosine ammonia-lyase; 4CL: 4-coumarin-CoA ligase; ACC: acetyl-CoA carboxylase; CHS: chalcone synthase; CHI: chalcone isomerase; PPO: polyphenol oxidase; F3H: flavanone 3-hydroxylase; IFS: isoflavone synthase; FNS: flavone synthase; DFR: dihydroflavonol 4-reductase; FLS: flavonol synthase; LAR: leucoanthocyanidin reductase; ANS: anthocyanidin synthase.

As a kind of secondary plant metabolite, the biosynthesis pathway of flavonoids in plants has been well studied. Understanding the biosynthesis of flavonoids is essential to enrich the resources of flavonoids and increase the production of flavonoids. Flavonoids are synthesized through the phenylpropanoid pathway [56]. The initial precursors of the phenylpropanoid pathway are L-phenylalanine or L-tyrosine, which are generally produced through the shikimate and aerogenate pathways [7]. The primary metabolite glucose produces phosphoenolpyruvate (PEP) and erythrose 4-phosphate (E4P) through the glycolytic pathway and pentose phosphate pathway, respectively, and they react to produce shikimic acid [57]. After a series of enzymatic reactions, shikimic acid can produce chorismate, prephenate, and finally L-phenylalanine and L-tyrosine [7] (Figure 2).

The phenylpropanoid pathway is a ubiquitous and well-described pathway for plant secondary metabolites. The first three common steps of the phenylpropanoid pathway are catalyzed by phenylalanine ammonia lyase (PAL), cinnamic acid 4-hydroxylase (C4H), and 4-coumarolyl-CoA ligase (4CL) [1]. Phenylalanine ammonia lyase (PAL) is responsible for converting L-phenylalanine into cinnamic acid, which is then oxidized by cinnamic acid 4-hydroxylase (C4H) to *p*-coumaric acid [56]. 4-coumarin-CoA ligase (4CL) converts *p*-coumaric acid into *p*-coumaroyl-CoA, which is one of the precursors of C6–C3–C6 backbone biosynthesis of different flavonoid classes [12]. However, the pathway of L-tyrosine conversion is shorter than the pathway using L-phenylalanine as the starting precursor. L-tyrosine can be directly catalyzed by tyrosine ammonia lyase (TAL) to *p*-coumaric acid, bypassing the C4H intermediate [12]. Then, a molecule of *p*-coumaroyl CoA and three molecules of malonyl-CoA are condensed to form naringenin chalcone catalyzed by chalcones synthase (CHS), which is an initial reaction in the synthesis of flavonoid classes in the phenylpropanoid pathway [7]. Malonyl-CoA is mainly produced by acetyl-CoA via the catalysis of acetyl-CoA carboxylase (ACC) [7]. Subsequently, the chalcone backbone is catalyzed by different classes of enzymes (such as isomerase, hydroxylase, oxido-reductase, transferase) to produce other flavonoid subgroups [8]. Chalcones can be converted into flavanones by chalcone isomerase (CHI) or to aurones by plant polyphenol oxidase (PPO) [8]. However, flavanones can not only be hydroxylated by flavanone 3−OH transferase (F3H) to produce flavanonols but can also be converted into flavones with a double bond between the C-2 and C-3 (of the C-ring) through the enzymatic action of flavone synthase (FNS) [58]. In addition, flavanones can also be converted into isoflavones via isoflavone synthase (IFS) [58]. Next, flavonol synthase (FLS) can oxidize flavanonols to flavonols, while dihydroflavonol-4-reductase (DFR) can reduce flavanonols to give rise to flavan-3,4-diols (also named leucoanthocyanidins) [58]. Finally, flavan-3,4-diols can be transformed into anthocyanidins by anthocyanidin synthase (ANS), and leucoanthocyanidin reductase (LAR) directly reduces flavan-3,4-diols to the corresponding flavan-3-ols [8]. These compounds are further modified into various flavonoids by glycosyltransferase, methyltransferase, acyltransferase, and so on [7].

## 4. Biosynthesis of Flavonoids by Metabolic Engineering

### 4.1. Microbial Mono-Culture for Flavonoid Synthesis

Recently, microbial fermentation methods for the high-yield production of natural plant products have been extensively investigated. Table 2 shows the applications of microbial mono-culture for the production of flavonoids in the past five years. As shown in Table 2, *E. coli* and *S. cerevisiae* are commonly selected microbial hosts. *E. coli* is usually the host of choice because it has several advantages, including a high growth rate, established high cell density culture technology, and extensive investigations in genetic engineering [59]. As a GRAS (generally regarded as safe) strain, *S. cerevisiae* is often used as a product host, and it has a complete intracellular membrane system and is superior to *E. coli* in terms of the expression of membrane proteins [57]. In the process of de novo synthesis of anthocyanins by *S. cerevisiae*, a higher titer of eriodictyol was obtained, which was 152 mg/L [60]. Eriodictyol was formed by the hydroxylation of naringenin by the cytochrome P450 (CYP) enzymes flavonoid-3-hydroxylase (F3′H). The high expression of F3ʹH demonstrated the superior ability of *S. cerevisiae* to functionally express plant CYPs. In this study, pelargonidin-3-O-glucoside (P3G), cyanidin-3-O-glucoside (C3G), and delphinidin-3-O-glucoside (D3G) were synthesized from glucose in *S. cerevisiae* for the first time, and their titers were 0.85, 1.55, and 1.86 mg/L, respectively [60]. When de novo biosynthesis of flavonoids is conducted by microorganisms, the efficient expression and targeting of pathway enzymes is the key to improving product yield. Apart from selecting suitable microbial hosts for heterologous expression of pathway enzymes, other strategies had been adopted. For example, when icariin was generated in *S. cerevisiae*, the pathway enzyme methyltransferase GmOMT2 would lose its activity due to the low pH in the cytoplasm if it was expressed in the cytoplasm [57]. In order to solve this problem, GmOMT2 could be relocated into the mitochondria (higher pH than cytoplasm) of *S. cerevisiae*. The synthesis of icaritin from glucose in microorganisms was also achieved for the first time, and the yield was 7.2 mg/L [57]. However, this regulation mechanism is still under elucidation for GmOMT2. The balance of biosynthetic pathways is another key to production optimization. In order to balance the metabolic flux, an iterative high-throughput balancing (IHTB) strategy was built to thoroughly fine-tune the naringenin biosynthetic pathway [22]. It constructed all possible gene-promoter combinations and then screened them many times. After several rounds of high-throughput screening, the metabolic flux of naringin synthesis pathway reached equilibrium, and the final titer was 191.9 mg/L [22].

Malonyl-CoA is an important precursor for the production of flavonoids, and three molecules of malonyl-CoA and one molecule of *p*-coumaroyl-CoA were mixed to synthesize naringenin chalcone by chalcone synthase. At the same time, it is also an essential intermediate for the synthesis of fatty acids that support cell growth in microorganisms [58]. In order to efficiently produce flavonoids, it is important to balance the biosynthesis of flavonoids and cell growth. Recently, an induction-free system to de novo biosynthesize naringenin from glucose by *E. coli* was established, and this system could automatically balance the production of naringenin, the accumulation of *p*-coumaric acid, and cell growth [61]. Because the direct precursor of malonyl-CoA is acetyl-CoA, the biosynthesis of acetyl-CoA is closely related to carbon source and acetic acid, so carbon source and acetic acid can be used to regulate the synthesis of acetyl-CoA and then the synthesis of malonyl-CoA. In order to increase the production of malonyl-CoA at the fermentation level, the concentration of glucose, glycerol, and potassium acetate (KAc) in the medium was optimized [61]. After optimization, the yield of naringenin could reach 588 mg/L, which was the highest titer reported in *E. coli* so far. In another study, a growth-coupled NCOMB (naringenin-coumaric acid-malonyl-CoA-balanced) DRN (dynamic regulation network) was developed, which could directly sense the accumulated naringenin and *p*-coumadin acid to dynamically regulate gene expression and finally achieve the goal of synchronizing cell growth and supplementing malonyl-CoA to form naringenin [62]. The yield of naringenin obtained by this method was 523.7 mg/L. During the biosynthesis of flavonoids, glucose, glycerol, and some amino acids (L-tyrosine and L-phenylalanine) are generally used as the substrates to synthesize flavonoids in *E. coli* or *S. cerevisiae*. Other microorganisms (*S. albus*, *Y. lipolytica*, *Corynebacterium glutamicum* (*C. glutamicum*), *Pichia pastoris* (*P. pastoris*), and *Lactococcus lactis* (*L. lactis*)) have also been studied as microbial hosts, and some intermediate products (naringenin, taxifolin, afzelechin, and catechin) in the biosynthetic pathways of flavonoids and natural products (tea) have also been used as substrates [2,20,63,64,65]. In recent reports, anthocyanins were generated from green tea by engineered *L. lactis* strains, and the total titer of anthocyanins was 1.5 mg/L [65]. Specifically, the engineered strain not only produced the expected red-purple compounds cyanidin and delphinidin, but also orange and yellow pyranoanthocyanidins with unexpected methylation patterns after fermentation.

**Table 2:** Microbial mono-culture for the production of flavonoids in the past five years.

Product	Substrate	Host Strain	Titer (mg/L)	Reference
Luteolin	Glucose	*S. albus*	0.09	[66]
Apigenin	Glucose	*S. albus*	0.089	[66]
Apigenin	Glucose and naringenin	*S. albus*	0.384	[66]
Scutellarin	Glucose	*S. cerevisiae*	108	[67]
Apigenin-7-O-glucuronide	Glucose	*S. cerevisiae*	185	[67]
Baicalein	L-phenylalanine	*E. coli*	23.6	[13]
Scutellarein	L-tyrosine	*E. coli*	106.5	[13]
Isoorientin	Luteolin	*E. coli*	3829	[68]
Isovitexin	Apigenin	*E. coli*	3772	[68]
Liquiritigenin	Glucose	*S. cerevisiae*	5.31	[16]
Eriodictyol	Glucose	*S. albus*	0.002	[66]
Eriodictyol	Naringenin	*E. coli*	62.7	[69]
Eriodictyol	Caffeic acid	*C. glutamicum*	37	[63]
Eriodictyol	Glucose	*S. cerevisiae*	152	[60]
Eriodictyol	Glucose	*Y. lipolytica*	54.2	[70]
Eriodictyol	Glucose	*Y. lipolytica*	134.2	[2]
Eriodictyol	Glycerol	*E. coli*	88	[19]
Homoeriodictyol	Glycerol	*E. coli*	17	[19]
Pinocembrin	Glycerol	*E. coli*	214	[19]
Naringenin	*p*-Coumaric acid	*C. glutamicum*	35	[63]
Naringenin	Tyrosine	*S. cerevisiae*	90	[71]
Naringenin	Tyrosine	*E. coli*	191.9	[22]
Naringenin	Glucose	*Y. lipolytica*	71.2	[70]
Naringenin	Glucose	*Y. lipolytica*	252.4	[2]
Naringenin	*p*-Coumaric acid	*S. cerevisiae*	648.63	[72]
Naringenin	Glycerol	*E. coli*	484	[19]
Naringenin	Glucose	*E. coli*	588	[61]
Naringenin	Glucose	*E. coli*	523.7	[62]
Fisetin	Glucose	*S. cerevisiae*	2.3	[16]
Resokaempferol	Glucose	*S. cerevisiae*	14.54	[16]
Kaempferol	Glucose	*S. cerevisiae*	26.57	[16]
Quercetin	Glucose	*S. cerevisiae*	20.38	[16]
Quercetin	Glucose	*S. albus*	0.599	[20]
Taxifolin	Glucose	*Y. lipolytica*	48.1	[70]
Taxifolin	Glucose	*Y. lipolytica*	110.5	[2]
Silybin and isosilybin	Eugenol and taxifolin	*E. coli*	2580	[73]
Taxifolin	Glucose	*S. cerevisiae*	336.8	[74]
Catechin	Afzelechin	*E. coli*	34.7	[69]
Peonidin 3-O-glucoside	Catechin	*E. coli*	56.3	[75]
Pelargonidin 3-O-glucoside	Glucose	*S. cerevisiae*	0.85	[60]
Cyanidin 3-O-glucoside	Glucose	*S. cerevisiae*	1.55	[60]
Delphinidin 3-O-glucoside	Glucose	*S. cerevisiae*	1.86	[60]
Cyanidin 3-O-glucoside	Catechin	*C. glutamicum*	40	[76]
Cyanidin 3-O-glucoside	Catechin	*E. coli*	439	[77]
Anthocyanins	Tea	*L. lactis*	1.5	[65]
Orobol	Genistein	*P. pastoris*	23	[64]
4ʹ-O-methyl-genistein	Genistein	*E. coli*	48.61	[78]
4ʹ-O-methyl-daidzein	Daidzein	*E. coli*	102.88	[78]
Icaritin	Glucose	*S. cerevisiae*	7.2	[57]

### 4.2. Microbial Co-Culture for Flavonoid Synthesis

Since long ago, humankind has used natural communities to ferment foods and beverages, produce drugs and chemicals, and treat wastes [79]. However, only recently have scientists begun to investigate the application of co-culture techniques in metabolic engineering and synthetic biology. Modular co-culture engineering is to cultivate two or more microbial strains to complete a target biosynthetic pathway. The complete biosynthetic pathway is firstly modularized, and then each microorganism is responsible for a specific module. The type of relationship between the cultured microbial hosts can be commensalism, cooperation, amensalism, predation, and no interaction [80]. Compared with the traditional mono-culture method, microbial co-culturing greatly reduces the biosynthetic labor of each strain, lowers the related metabolic burden, and withstands greater environmental disturbances. Currently, microbial co-culture techniques have been applied for the production of various compounds, including fuels, amino acids, proteins, polyphenols (including flavonoids), alkaloids, terpenoids, and other chemicals [80,81].

Due to the clarification of the biosynthetic pathway of flavonoids, characterization of pathway enzymes, and wide application of flavonoids, flavonoids are more attentioned natural products through modular co-culture engineering biosynthesis, including genistein, naringenin, apigetrin, pelargonidin 3-O-glucoside, icaritin, and (+)-afzelecin, compared with other natural products (such as alkaloids and terpenoids) [81]. As shown in Table 3, co-cultured strains are mostly different strains of the same organism, which may be due to the fact that the use of different strains within the same organism in multi-culture can simplify growth factors, antibiotics, and downstream processing. Microbial co-culture technology was employed for the production of flavonoids starting in 2007 [82]. The co-culture of *S. cerevisiae* carrying the IFS gene and *E. coli* with a high ability to produce (S)-naringenin (the direct precursor of genistein) from tyrosine could produce genistein. The yield of genistein was 6 mg/L, which was 17.6 times higher than that of genistein produced by mono-culture of *S. cerevisiae* (0.34 mg/L) [82]. This is mainly due to the reduction of metabolic stress and the use of an appropriate host to increase pathway enzyme activity. After optimization of co-culture conditions, the titer of genistein was increased to 100 mg/L [83]. Naringenin is the central precursor of most flavonoids, and its synthesis by co-culture using different substrates has been reported. When naringenin was synthesized de novo from glucose through the *E. coli–E. coli* co-culture system, the biosynthesis pathway of naringenin was split into two modules [84]. An *E. coli* strain (known as the upstream strain) was designed to convert glucose into tyrosine or *p*-coumaric acid (module 1), and the other *E. coli* strain (known as the downstream strain) was constructed to be responsible for the conversion of tyrosine or *p*-coumaric acid to naringenin (module 2). The relationship between the two strains was commensalism. Meanwhile, some strategies, including screening *E. coli* strains and optimizing IPTG induction time and the inoculation ratio of co-culture strains, were adopted to adjust and balance the biosynthetic intensity between different pathway segments. Finally, the yield of naringenin was increased to 41.50 mg/L. In addition, naringenin has been synthesized by the co-culture of *E. coli* and *S. cerevisiae*, and co-culture of *S. cerevisiae* and *S. cerevisiae* using xylose and *p*-coumaric acid as substrates, respectively [85,86]. However, the titers were lower than that of naringenin produced by the co-culture of *E. coli* and *E. coli*. Notably, the co-culture of *S. cerevisiae* and *S. cerevisiae* strains separately containing AlCHS and Al4CL could produce not only naringenin but also resveratrol [86]. An artificial icaritin biosynthetic pathway was initially designed to generate icaritin in microbes this year [57]. During the biosynthesis of icariin, the role of methyltransferase GmOMT2 was very important. GmOMT2 was sensitive to low pH and loses its activity when expressed in yeast cytoplasm; this was the widespread issue of incompatible pH conditions encountered in basic and applied bioproduction research. In this study, an *E. coli* strain expressing GmOMT2 and the 8-prenylkaempferol-producing *S. cerevisiae* strain were co-cultured to overcome the difficulty. 8-Prenylkaempferol-producing yeast strain was constructed to efficiently generate 8-prenylkaempferol from glucose via introducing 11 heterologous genes and modifying 12 native yeast genes. The yield of icaritin produced by co-culturing *S. cerevisiae* and *E. coli* was 19.70 mg/L, which was higher than that of mono-culture *S. cerevisiae* (7.20 mg/L, as described in 4.1) [57]. By dividing the long synthetic pathway into different microbial strains, it provides a better environment for specific enzymes or the overexpression of some pathway genes, minimizes feedback inhibition, and reduces the pressure of metabolic flux.

In addition to free aglycones being synthesized by co-culture strategy, some glycosylated flavonoids have also been successfully synthesized through synthetic microbial consortia. Different from free flavonoids, the synthesis process of glycosylated flavonoids needs to introduce some glycosyltransferases to modify these compounds and provide sufficient sugar groups. For example, co-culture systems were developed in two *E. coli* strains to produce apigetrin (Apigenin-7-O-b-D-glucoside). An upstream strain containing 4CL from *Nicotiana tabacum*, CHS from *P. hybrida*, CHI from *M. sativa*, and FNS from *Parsley* specially synthesized apigenin from *p*-coumaric acid, and the downstream strain was responsible for the overproduction of UDP-glucose and heterologous expression of a glycosyltransferase (PaGT3) to convert apigenin into apigetrin [87]. By optimizing the initial inoculum ratio of strains, temperature, and media component, the titer of apigetrin could reach 16.60 mg/L. Meanwhile, this co-culture strategy restricted the formation of undesired byproducts. Another typical example is the de novo biosynthesis of pelargonidin-3-O-glucoside by the mixed culture of four different *E. coli* strains. De novo biosynthesis of pelargonidin-3-O-glucoside is difficult to be realized because of its complicated biosynthesis process and too many enzymes. In previous studies, pelargonidin-3-o-glucoside was successfully synthesized from naringin using two different engineered *E. coli*, with yields of 5.6 and 78.9 mg/L, respectively [88,89]. In these studies, pelargonidin-3-o-glucoside was not synthesized de novo. Recently, de novo biosynthesis of pelargonidin-3-O-glucoside has been successfully achieved by *S. cerevisiae*, but the yield was low, only 0.85 mg/L [60]. In the co-culture system, 15 enzymes and transcriptional factors were overexpressed on four independent *E. coli* strains, and the pelargonidin-3-O-glucoside synthesis pathway was divided into four modules [90]. The first strain was responsible for converting glucose, xylose, and glycerol into phenylpropionic acid. The second strain utilized phenylpropionic acid and exogenously added malonate to generate flavanones, which were transformed into flavan-3-ols through the third strain. The fourth strain overexpressed anthocyanin synthase (ANS) and 3-O-glycotransferase (3GT) genes to convert flavane-3-ols to anthocyanidins, and glycosylation modification of anthocyanidins, respectively. When using the synthetic microbial consortia to synthesize pelargonidin-3-O-glucoside, the optimum yield was 9.5 mg/L, which was 11.2 times that of the mono-culture. These linear, binary, ternary, and even quaternary designs are used in more complex linear biosynthetic pathways to reduce the metabolic burden of each strain and enable independent strains to optimize modules for specific needs. Other examples of using a co-culture system to synthesize flavonoids are also summarized in Table 3.

**Table 3:** Recent examples of modular co-culture engineering for flavonoids production.

Product	Substrate	Co-Culture System	Titer (mg/L)	Reference
Genistein	Tyrosine	*E. coli*–*S. cerevisiae* coculture	6	[82]
Genistein	Tyrosine	*E. coli*–*S. cerevisiae* coculture	100	[83]
(+)-Afzelechin	*p*-Coumaric acid	*E. coli*–*E. coli* coculture	40.7	[21]
(+)-Afzelechin	Glucose, glycerol and xylose	Three-strain *E. coli* polyculture	26.1	[90]
Naringenin	Glucose	*E. coli*–*E. coli* coculture	41.5	[84]
Naringenin	*p*-Coumaric acid	*S. cerevisiae*–*S. cerevisiae* coculture	18.5	[86]
Naringenin	Xylose	*E. coli*–*S. cerevisiae* coculture	21.16	[85]
Pelargonidin 3-O-glucoside	Glucose, glycerol and xylose	Four-strain *E. coli* polyculture	9.5	[90]
Apigetrin	*p*-Coumaric acid	*E. coli*–*E. coli* coculture	16.6	[87]
Pyranocyanidin-3-O-glucoside-phenol	Glucose, (+)- catechin and tyrosine	*E. coli*–*E. coli* coculture	19.5	[91]
Pyranocyanidin-3-O-glucoside-catechol	Glucose and (+)- catechin	*E. coli*–*E. coli* coculture	13	[91]
Sakuranetin	Glucose	*E. coli*–*E. coli* coculture	79	[92]
Vitexin	Luteolin and apigenin	*E. coli*–*E. coli* coculture	5050	[93]
Orientin	Luteolin and apigenin	*E. coli*–*E. coli* coculture	7090	[93]
Icaritin	Glucose	*E. coli*–*S. cerevisiae* coculture	19.7	[57]

## 5. Conclusions

In recent times, the continuous development of synthetic biology and basic genetic engineering has not only increased the type and number of microbial hosts used to synthesize flavonoids but also increased the titer of some flavonoids. Likewise, modular co-cultivation engineering has gradually been studied for the synthesis of some natural products as an emerging method. It has some advantages in synthesizing natural products with longer biosynthetic pathways and more complex structures. At present, some flavonoids have been synthesized by co-culture strategy. Microbial co-culture strategy provides a new direction to improve the production efficiency of flavonoids and controls production cost.

## Figures and Tables

**Table 1:** Structural features and classification of flavonoids.

Groups		Structures	Examples
Flavones		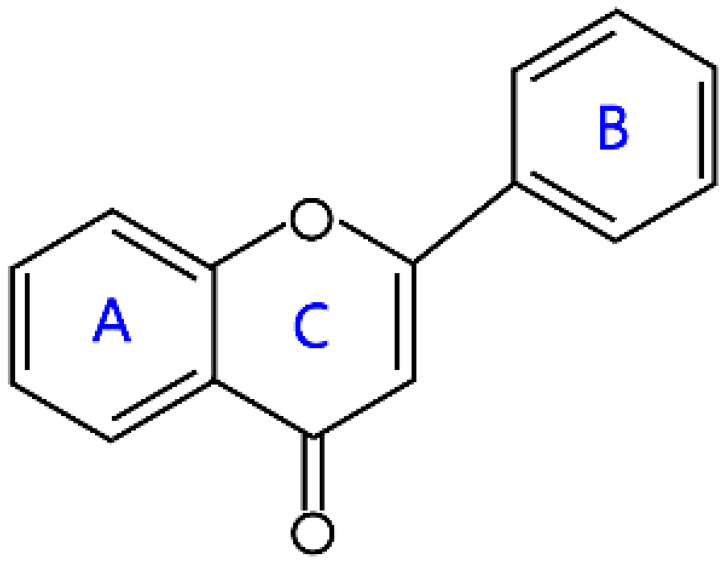	Apigenin, luteolin, baicalein, and tangeritin
Flavanones		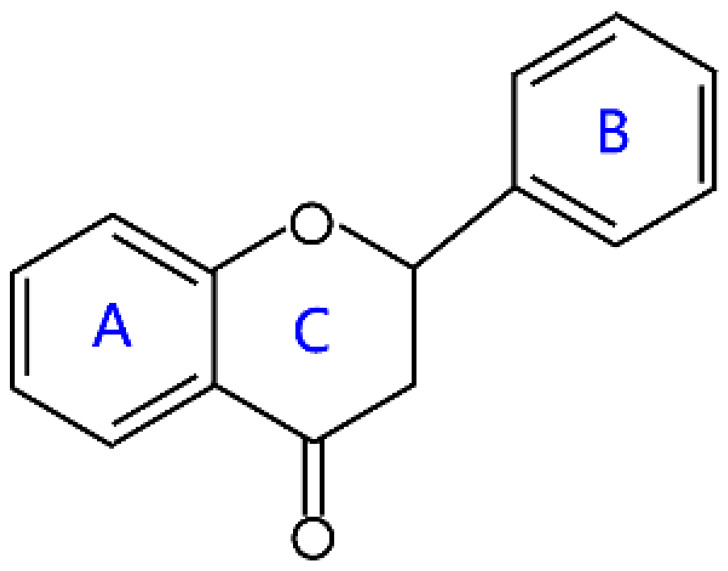	Liquiritigenin, hesperitin, naringenin, and eriodictyol
Flavonols		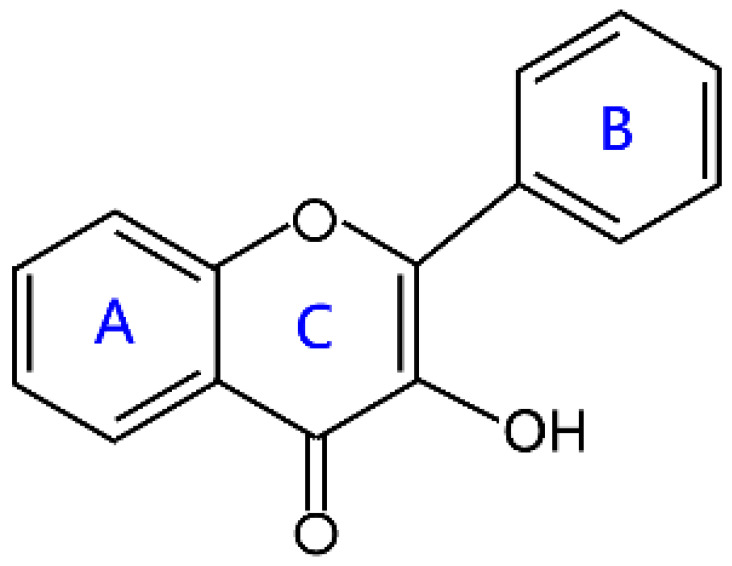	Kaempferol, quercetin, myricetin, and rutin
Flavanonols		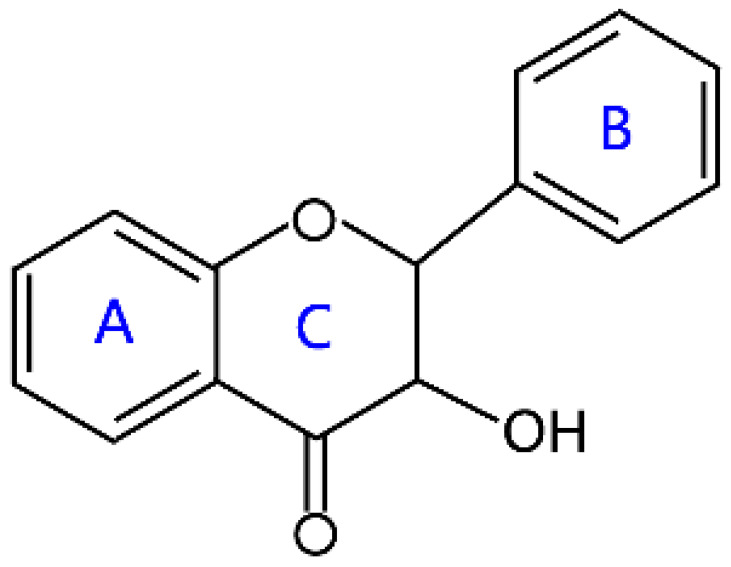	Aromadendrin, distylin, dihydrokaempferol, and dihydroquercetin
Flavanols	Flavan-3-ols	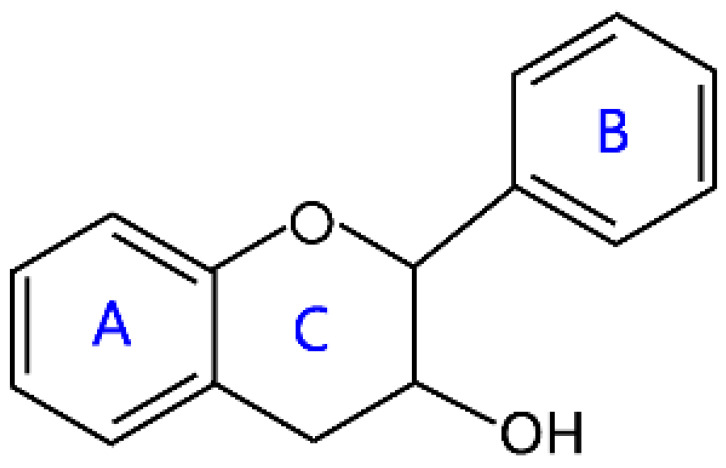	Catechin, elephantorrhizol, guibourtinidol, and epirobinetinidol
	Flavan-3,4-diols	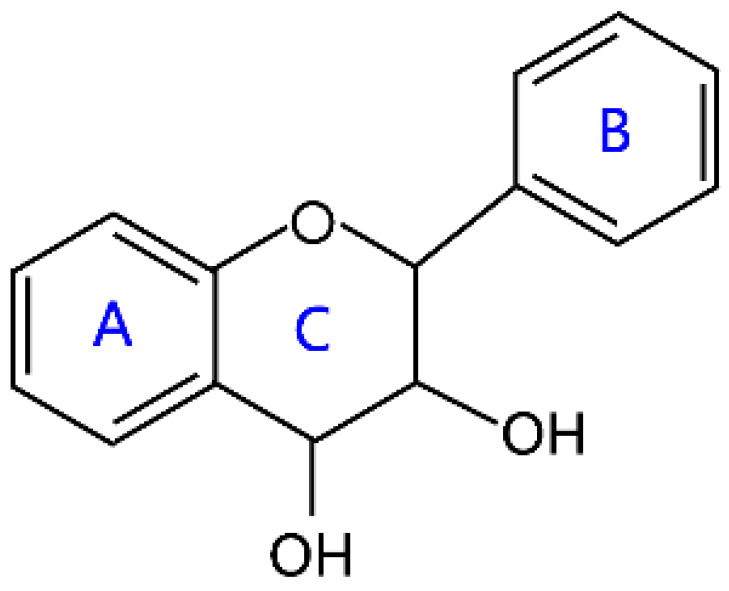	Leucofisetinidin, leucocyanidin, melacacidin, and leucopeonidin
Anthocyanidins		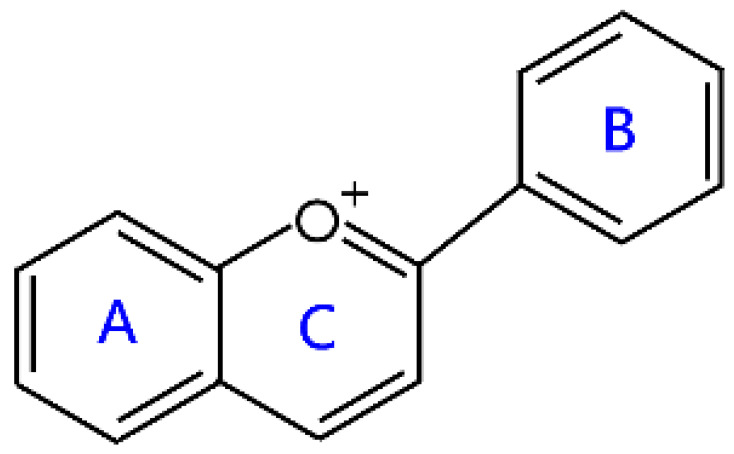	Cyanidin, delphinidin, malvidin, peonidin, pelargonidin, and petunidin
Chalcones		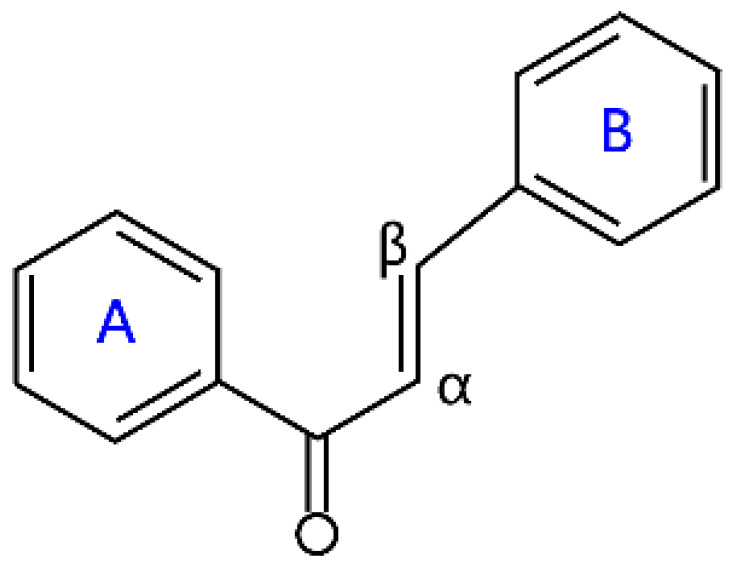	Naringenin chalcone, pinocembrin chalcone, echinatin, and licochalcone A
Dihydrochalcones		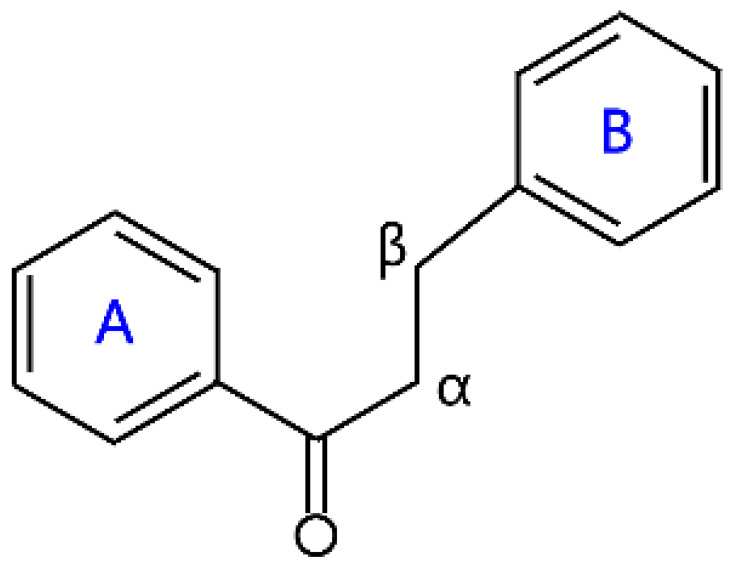	Neohesperidin dihydrochalcone, phloridzin, phloretin, and naringin dihydrochalcone
Aurones		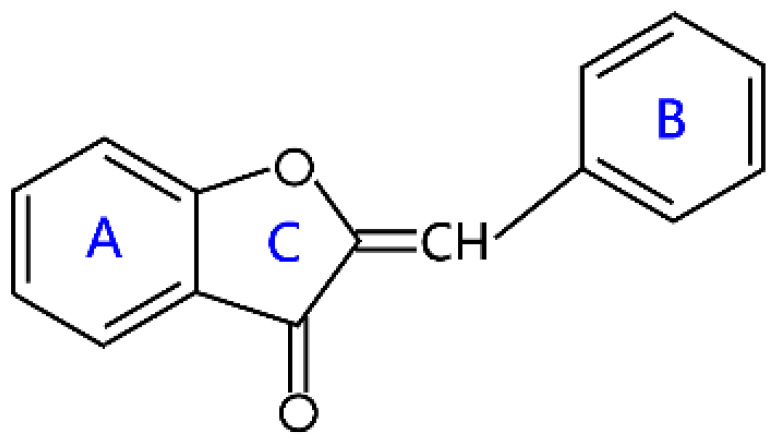	Aureusidin, maritimetin, hispidol, and hamiltrone
Isoflavonoids	Isoflavones	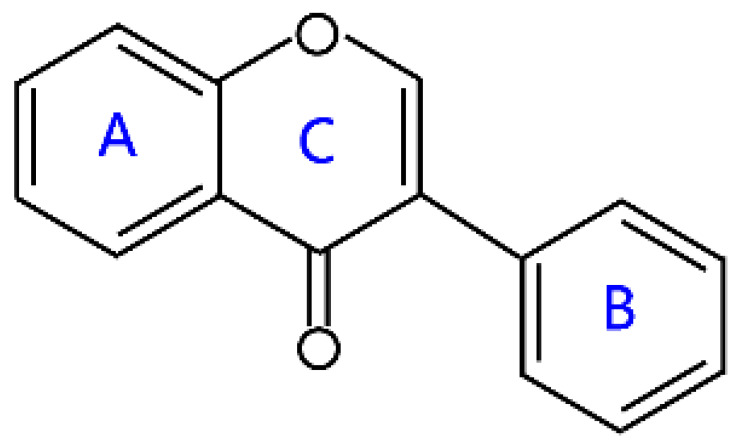	Daidzein, genistein, biochanin A, glycitein, and formononetin
	Isoflavanones	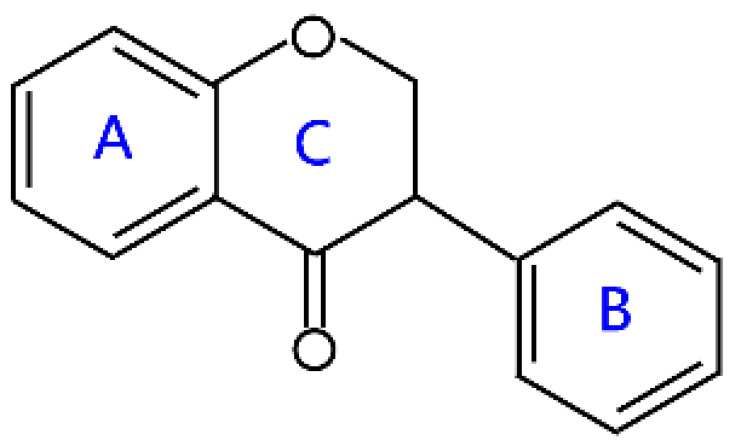	Pterostilbene, trifolium sophorin, and pterostilbene
	Isoflavans	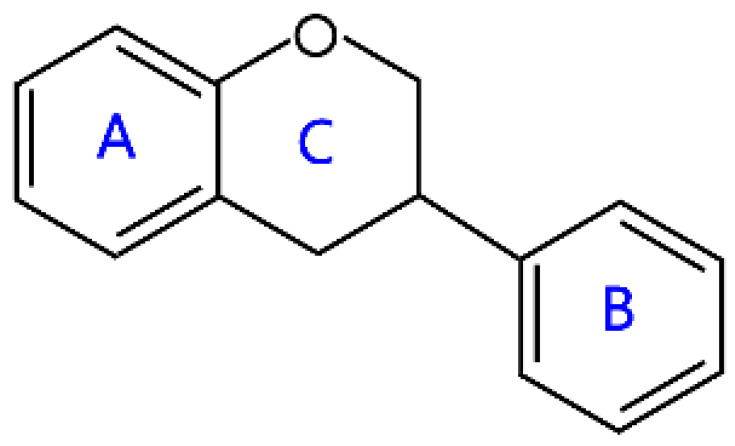	Equol
Neoflavonoids		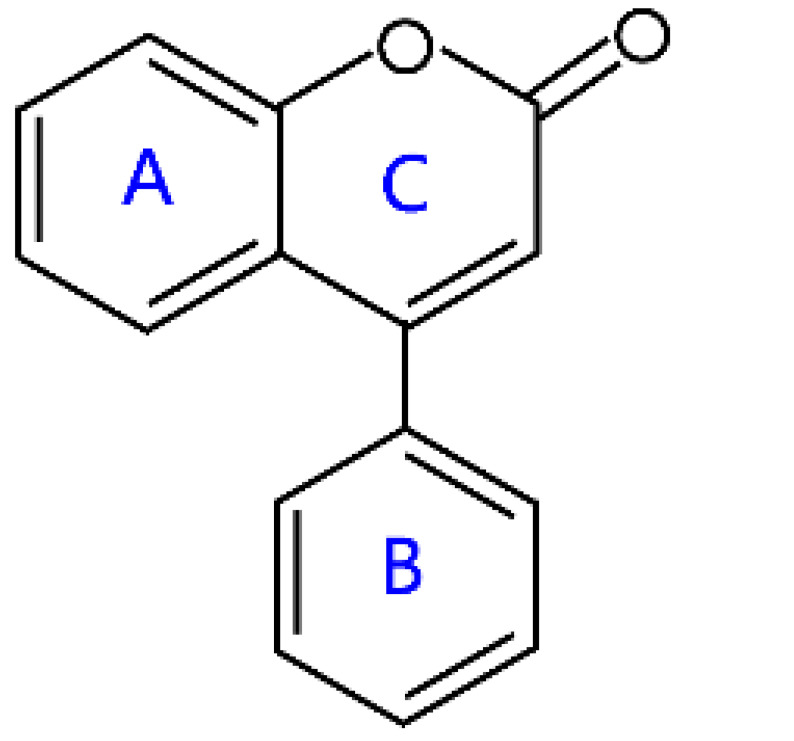	Dalbergin and calophyllolide

## Data Availability

Data sharing not applicable. No new data were created or analyzed in this study.
